# Mysteries of Tobacco

**Published:** 1846-02

**Authors:** 


### MYSTERIES OF TOBACCO.

This is the title of a little work published by Wiley & Putnam of New York, from the pen of the Rev. Benjamin I. Lane, and although claiming a paternity other than medical, it comes within the purview of the medical critic. It is an argument against tobacco, and in several chapters discusses the nature of "the nauseous weed," its influence upon the body, the mind, and the morals; the illusory influence of tobacco, its filthiness, and its expensiveness. But *cui bono?* "To write against tobacco, with its mysteries and its luxuries," is, indeed, "an unpromising business." The author of this little book feels it to be so, but he is nerved with the hope of doing good, and so "raises the note of warning, and spares not." He gives, first, the analysis of tobacco; then some experiments by Fontana upon animals showing its poisonous character, and, lastly, its medicinal qualities. In the chapter on the physical effects of tobacco, he cites a cloud of witnesses to prove its injurious influence upon health. Its influence upon the mind he thinks is also pernicious; for he does not agree with the physician who was asked by a lady, whether he thought snuff injured the brain of those who use it, and who replied, "no, for nobody that has any brains uses it." This is not quite true; but it is a fact, he adds, "worthy of note in the history of tobacco, that its use began among the ignorant savages."

The fault of this well-meant volume is, that the picture drawn in it of the mischief done by tobacco is exaggerated. The author promised "nothing to extenuate, nor set down aught in malice," and the truth is certainly revolting enough; but he has not stuck to his promise. He has not been satisfied to show the evils of chewing and smoking in their full natural size—he has held up to the eyes of his readers a magnifying glass, by which their proportions become, not hideous, but ridiculous. Still, we wish every smoker and chewer would read the "Mysteries of Tobacco," that they might see how the indulgence appears to, at least, one sensible, eloquent, and learned man. Or rather, we wish, with Mr. John Quincy Adams, to whom the volume is dedicated, who was once addicted to the use of tobacco, "that every individual of the human race afflicted with this artificial passion, could prevail upon himself to try but for three months the experiment which he made, sure that it would turn every acre of tobacco-land into a wheat-field, and add five years of longevity to the average of human life."

We thank the publishers for this spirited tract, which, with several other volumes to be noticed, we have received through Messrs. Maxwell and Prescott from the same publishers.

